# Physical exercise prepartum to support metabolic adaptation in the transition period of dairy cattle: A proof of concept

**DOI:** 10.1111/jpn.13330

**Published:** 2020-02-25

**Authors:** Roselinde M. A. Goselink, Jan Thomas Schonewille, Gert van Duinkerken, Wouter H. Hendriks

**Affiliations:** ^1^ Department of Animal Nutrition Wageningen Livestock Research Wageningen the Netherlands; ^2^ Faculty of Veterinary Medicine Department of Farm Animal Health Utrecht University Utrecht the Netherlands; ^3^ Chair Group of Animal Nutrition Wageningen University Wageningen the Netherlands

**Keywords:** body condition, calving, dairy cow, energy metabolism, exercise physiology, fat metabolism, hepatic lipidosis, lipoprotein

## Abstract

In dairy cattle, the hormonal changes around calving induce large metabolic changes to support milk production. Mobilization of adipose reserves is one of the changes involved, imposing a metabolic load on the liver. We hypothesized that the risk for excessive lipolysis and hepatic lipidosis postpartum can be reduced by starting fat mobilization and processing during the prepartum period by physical exercise, especially in cows with a high body condition score (BCS). As a proof of concept, 32 pregnant Holstein‐Friesian dairy cows were selected for a 2 × 2 experimental design. Sixteen cows had a BCS < 3.25 (group LOW) and 16 cows a BCS ≥ 3.25 (group HIGH). Cows within each group were randomly allocated to one of two treatments: group STEP was walked twice daily for 45 min during the dry period while group CON remained indoors. Treatment was stopped at calving and cows were monitored until 6 weeks after calving. Liver biopsies were taken in a subset of 16 cows to determine liver triglyceride (TG) concentration. We found that calculated energy balance was more negative for group STEP prepartum, resulting in higher plasma non‐esterified fatty acids and β‐hydroxybutyrate concentrations. During the first 6 weeks postpartum, neither dry matter intake nor milk yield was affected by exercise. As expected, the cows in group HIGH had increased liver TG concentrations postpartum relative to group LOW with increased plasma non‐esterified fatty acids directly after calving. Exercise during the dry period mitigated postpartal liver TG accumulation, but this did not seem to be related to increased plasma lipoprotein transport. We conclude that substantial physical activity prepartum can induce lipolysis and lipid utilization, thereby starting an early adaptation to lactation. This may be instrumental to reduce the risk for excessive liver TG accumulation postpartum, especially in cows with a high BCS at dry‐off.

## 1 INTRODUCTION

Hormone‐induced adaptations in fat metabolism lead to increased fat mobilization in dairy cows around calving, thereby leading to an increased release of fatty acids from adipose tissue elevating blood non‐esterified fatty acids (NEFA) concentrations (Friggens, Andersen, Larsen, Aaes, & Dewhurst, 2004). This release can be considered an evolutionary advantage to provide energy for milk synthesis for the survival of the offspring. At the same time however, the risk for hepatic lipidosis is increased (Van Knegsel et al., 2014).

Important risk factors associated with hepatic lipidosis are overfeeding during the dry period and a high body condition score (BCS) at calving (Roche et al., 2015; Van Den Top et al., 1995). It is generally accepted that a high BCS (>3.5) at calving causes excessive mobilization of body fat postpartum, thereby increasing the risk for metabolic diseases such as ketosis and fatty liver (Gillund, Reksen, Gröhn, & Karlberg, 2001; Roche et al., 2015). Avoiding excess energy intake during the dry period is therefore an important strategy to prevent excessive fat mobilization postpartum (Janovick, Boisclair, & Drackley, 2011; Roche et al., 2015). An alternative strategy may be to trigger fat mobilization and early adaptation of metabolic processes needed postpartum by inducing a mild negative energy balance (NEB) before calving. This may be achieved by increasing physical activity during the dry period as a method to increase energy output and stimulate energy metabolism. Besides the net energy cost of exercise, training will also stimulate lipolysis of adipose tissue while enhancing muscular development and muscle enzyme activity, as described in humans (Tremblay, Simoneau, & Bouchard, 1994). The outcome of previous research indicates that prepartal exercise of 1.6 up to 9.7 km/day reduces weight gain in multiparous cows, while feed intake and postpartal milk yield remain unaffected; nutrient intake and calculated energy balance were however not available (Anderson, Lamb, & Walters, 1979; Lamb, Anderson, & Walters, 1981). Walking 0.5 to 3 km/day did improve general health of tied dairy cows compared with non‐exercised, tied dairy cows, especially regarding disease incidence in the first two weeks after calving (Gustafson, 1993). Forcing dry cows to exercise for 1.25 to 1.5 hr/day at a speed of 3.25 km/hr resulted in improved physical fitness, as determined by a lower heart rate, lower plasma lactate and an improved ability to maintain their systemic acid–base balance at a given workload (Davidson & Beede, 2009).

In this study, we aimed to induce prepartal lipolysis using increased physical activity in dairy cows with normal vs. high BCS as a proof of concept. We hypothesize that especially in cows with a high BCS at dry‐off, prepartal physical exercise prevents excessive fat accumulation in the liver after calving.

## 2 MATERIALS AND METHODS

### 2.1 Animals and treatments

All experimental protocols and interventions were approved by the Ethical Committee on Animal Experiments of the Animal Sciences Group of Wageningen University & Research, Wageningen, the Netherlands. The experiment was conducted between April and October 2012 and comprised 32 pregnant Holstein‐Friesian cows selected at our 450‐cow research dairy farm in Lelystad, the Netherlands. The animals were selected based on parity (no heifers), expected calving date and BCS. The herd's mean BCS during the last trimester of gestation was 3.2 which was used as cut‐off level to define our study groups: 16 cows were selected with a BCS < 3.25 (group LOW) and 16 cows a BCS ≥ 3.25 (group HIGH) between 6 and 8 weeks before the expected calving date. The 32 selected cows were then blocked (two LOW and two HIGH cows per block) by parity, previous 305‐day milk production and expected calving date. Within each block, the two LOW cows were randomly allocated to either control (CON) or exercise (STEP) group and the same was done for the two HIGH cows.

The experiment started at dry‐off, 41 days (*SD* ± 7 days) before calving. The cows allocated to STEP received physical exercise during the entire dry period by walking two times per day (at 0730 and 1600 hr) for 45 min in a mechanical horse walker at a speed of 3.4 km/hr and an ambient temperature varying between 7 and 27°C. Exercise was stopped as soon as pelvic ligaments were weakened before calving (on average 1 day prior), and all cows were monitored for 6 weeks after calving.

Cows were housed in a cubicle shed with 1 cubicle available per cow and were kept in separate dry cow and post‐calving groups with approximately 5 m^2^ walking area per cow, without any access to either a pasture or outdoor paddock. At the first signs of calving, cows were separated from the dry cow group and housed in a straw bedded calving pen (20 m^2^) where the animals had unrestricted access to the dry cow diet and freshwater. Directly after calving, the cow was moved to the post‐calving group.

### 2.2 Rations and feeding management

Fresh feed mixtures were provided daily between 1030 and 1100 hr. From 6 week before until calving, cows received a dry cow feed mixture supplemented with 1.0 kg compound concentrate from 2 weeks before calving (Tables 1 and 2). During the prepartal period, daily energy intake was restricted to maximally 60 MJ of NE_L_/cow for both CON and STEP to prevent excessive gain of body fat (CVB, 2012).

**Table 1:** Ingredients of the feed mixtures and amounts of concentrate fed during pre‐ and post‐calving period of dairy cows

	Dry cow ration	Lactation ration
Feed mixture (% of DM)		
Wilted grass silage	46.7	51.9
Corn silage	15.8	33.3
Chopped rapeseed straw	2.8	3.1
Chopped wheat straw	23.6	‐
Rapeseed extract	9.9	3.1
Soya bean extract	‐	10.1
Vitamin and mineral premix	1.0	1.0
Magnesium oxide	0.2	‐
Sodium bicarbonate	‐	0.6
Concentrate (kg/cow/day)		
Barn^a^	0 to 1.0	1.0 to 8.5
Milking parlour^b^	‐	1.0

a Compound concentrate (based on ground corn, palm kernel expeller, rapeseed solvent extract, soy hulls and soya bean solvent extract) with 6.7 MJ NE_L_ and 180 g CP per kg; pre‐calving gradually increasing from 0 kg/day at day −14 to 1.0 kg/day on the expected day of calving, and post‐calving gradually increasing from 1.0 kg/day at day 0 to 8.5 kg/day at day 17 and maintained at 8.5 kg/day until the end of the trial.

b Compound concentrate (based on ground corn, palm kernel expeller, citrus pulp, rapeseed solvent extract and rapeseed formaldehyde treated) with 6.6 MJ NE_L_ and 165 g CP per kg.

**Table 2:** Chemical composition and feeding value of dry period feed mixture, lactation feed mixture and concentrate (g/kg of dry matter)

	Dry cow mixture	Lactation mixture	Concentrate
Chemical composition			
DM (g/kg of product)	526	458	878
Crude protein	117	149	206
Crude fat	31	31	38
NDF	544	430	298
ADF	318	246	166
Starch	54	110	220
Sugars	55	62	85
Ash	86	85	73
Feeding value			
DVE^a^	44	71	123
OEB^b^	18	29	28
FOM^c^	452	509	539
NE_L_ (MJ/kg of DM)	5.25	6.20	7.62

a Intestinal digestible protein (Tamminga et al., 1994).

b Rumen degraded protein balance (Tamminga et al., 1994).

c Fermentable organic matter.

Maximum energy intake was estimated to be ~110% of the mean (32 cows) energy requirement for maintenance and gestation during the last 6 weeks of gestation and did not include the energy requirement for exercise. Cows in group CON did not have access to the feeding bins during the periods cows in group STEP were outside exercising, to prevent a difference between the two treatment groups in the time available for eating.

After calving, all cows were fed the lactation feed mixture ad libitum (Tables 1 and 2). The cows were fed two compound concentrates: one supplied at milking (0.5 kg per milking, at 0600 and 1700 hr) and one in the barn (gradually increased with 0.5 kg/day from 1.0 kg/day on the day of calving up to 8.5 kg/day on day 17). The maximum level of concentrate was maintained from day 17 until the end of the experimental period at day 42.

Throughout the entire study, the cows had access to troughs with individual transponder‐controlled access gates (Roughage Intake Control system, Hokofarm Group, Marknesse, the Netherlands). The troughs were equipped with an electronic balance and allowed automatic registration of individual feed intakes. Except during exercise sessions (from 0730 to 0830 hr and from 1600 to 1700 hr) and feeding time (from 1030 to 1100 hr), cows had continuous access to the feed troughs. Overall, the amount of feed mixture that was supplied to the cows was 110% of actual intake. The compound concentrates were fed individually, using transponder‐controlled concentrate dispensers which made the individual allowance available in equal portions over six 4‐hr periods. All cows had unrestricted access to freshwater, except cows in group STEP during their physical training sessions in the horse walker.

### 2.3 Feed sampling and feed analysis

Feed mixtures were sampled daily for DM analysis. The DM content was determined after oven drying at 104°C during 36 hr. Each separate ingredient of the feed mixtures as well as the compound concentrates was sampled weekly and stored at −20°C. For grass and maize silage, samples of 5–6 consecutive weeks were pooled into 1 composite sample; for concentrates and wheat straw, all samples were pooled per feedstuff. The pooled samples were used for chemical analysis and determination of the feeding values at the MasterLab Laboratory (MasterLab, Boxmeer, the Netherlands). Briefly, the crude fat content was determined gravimetrically as the ether extract (ISO 6492; ISO, 1999) and crude ash content after incineration at 550°C (ISO 5984; ISO, 2002). Content of NDF and ADF were determined according to Van Soest, Robertson, and Lewis (1991) and expressed without residual ash. Crude protein was calculated as 6.25 × N‐Kjeldahl (ISO 5983; ISO, 2005), and sugar concentrations were determined as described by Van Vuuren, Van der Koelen, Valk, and De Visser (1993). Starch was released by heating in a boiling water bath in the presence of 2 NHCl (Cone, 1991) after which starch concentration was determined using the amyloglucosidase method (ISO 15,914; ISO, 2004). The NE_L_ and intestinal digestible protein (DVE) were calculated according to the guidelines from the Central Bureau for Livestock Feeding (CVB, 2012).

### 2.4 Milk yield and milk composition

Cows were milked twice daily at 0600 and 1700 hr with milk weights recorded automatically at each milking. Weekly, milk samples of each cow were taken at 4 consecutive milkings (two morning milkings and two afternoon milkings). Both morning milk samples were pooled to one composite sample; afternoon milk samples were processed likewise. The composite morning and afternoon milk samples were analysed for fat, protein and lactose concentrations by Qlip using a Foss MilkoScan infrared automatic analyzer (Foss Electric). Weighted means were calculated from the recorded morning and afternoon milk weights and the analyses of the composite morning and afternoon milk samples.

### 2.5 Body weight, body condition score and energy balance

The pre‐calving BW was recorded weekly between 0800 and 1000 hr before fresh feed was supplied. The post‐calving BW was automatically recorded at the same weighing scale, twice per day at entry into the milking parlour. Cows were scored weekly for body condition from 1 (thin) to 5 (fat) with 0.25 point increments according to Ferguson, Galligan, and Thomsen (1994). Energy balance was calculated as described by Van Knegsel et al. (2007) as the difference between net energy for lactation (NE_L_) supplied with feed and the NE_L_ required for gestation, maintenance and milk production. Calculations were based on the stage of gestation, average feed intake, milk yield, milk composition and body weight results of that week. The additional metabolizable energy (ME) requirement for forced walking at 3.4 km/hr is on average 0.52 kcal ME kg^‐1^km^−1^ (Hall & Brody, 1934), and as such for the present trial, energy required was estimated to be 4.6 MJ NE/day for cows of approximately 700 kg BW walking 5 km per day (ME: NE_L_ equivalent to 1:0.6).

### 2.6 Blood collection and analysis

Blood samples were taken twice prepartum, at 6 weeks (30–48 days) and 2 weeks (3–20 days) before calving and postpartum on day 3, 7, 10, 14, 21 and 42. Blood samples were collected from the tail vein (vena cauda) using lithium heparin‐coated tubes (Vacuette, type 455084, Greiner Bio‐One) for analyses of beta‐hydroxybutyrate (BHB) and in EDTA‐coated tubes (Vacuette, type 455036, Greiner Bio‐One) for analysis of NEFA, triglycerides (TG), high‐density lipoprotein (HDL) cholesterol and total cholesterol. Immediately after collection, blood samples were placed in ice water and centrifuged at 1,500 ***g*** for 10 min within 1 hr after collection. Subsequently, 1 ml of blood plasma was transferred to a vial and stored at −20°C until analysis.

NEFA were determined using a colorimetric assay and BHB by an enzymatic method (both kits from Randox Laboratories Ltd). Plasma total cholesterol and TG were measured using a commercial enzymatic dry chemistry kit (Ortho Clinical Diagnostics). After precipitation of low‐density lipoprotein (LDL) and very low‐density lipoprotein (VLDL) with sodium phosphotungstate magnesium chloride, HDL cholesterol was determined using a commercial enzymatic kit (Roche Diagnostics). Finally, LDL cholesterol was calculated as follows (all measures in mg/dl):

LDL cholesterol = total cholesterol−HDL cholesterol−TG/5.

where 5 is the ratio between TG and VLDL cholesterol (Friedewald, Levy, & Fredrickson, 1972).

### 2.7 Liver biopsies and analysis

Liver biopsies were taken from 16 cows (from 4 randomly selected blocks) in week−2 (6–23 days before calving), week 1 and week 2 relative to calving according to the method described by Zom et al. (2011). Briefly, a skin incision was made at the 11th intercostal space after local anaesthesia and a biopsy needle was inserted to collect ~2 g of liver tissue for analysis. Samples were immediately frozen in liquid N and stored at −80°C until analysis. Before analyses, liver samples were thawed and adhered water was removed using paper tissues. The concentrations of liver TG were determined using enzymatic hydrolysis of triglycerides with lipase into glycerol and fatty acids using the Triglycerides LiquiColor Mono test kit (Instruchemie B.V.) by photometric analysis at 550 nm (HumaLyzer 3000, Human Diagnostics).

### 2.8 Calculations and statistical analysis

Daily feed intake, milk yield, BW and energy balance were averaged per cow per week relative to calving. Liver biopsies were available for a subset of 16 cows (from four randomly chosen blocks) in week −2, week 1 and week 2 relative to calving; liver TG content was determined relative to wet weight and expressed as a relative value compared with the TG content in week −2, where the value of week −2 was set at 100%.

A mixed model analysis with repeated measures was performed using the REML procedure in Genstat 19th edition (2018). For the dry period and lactation period separately, each parameter was modelled over time for each individual cow using a random coefficient regression model, that is.

*Y*_ijkl_ = *μ *+ ε_i_ + *C*_j _+ *T*_k_ + *W*_l _+ *C*_j_ × *T*_k _+ *C*_j_ × *W*_l _+ *T*_k_ × *W*_l _+ *C*_j_ × *T*_k _× *W*_l_ + *C*_j _× *T*_k_ + ε_i·l_.

where *Y*_ijkl_ = dependent variable; *µ* = overall mean; ε_i_ = random effect of cow (where ε_i_ ~ *N*(0, $\sigma_{i}^{2}$); C_j_ = effect of BCS class (LOW or HIGH); *T*_k_ = effect of treatment (CON or STEP); W_l_ = effect of time (prepartum −6 to −1; postpartum 1 to 6); *C*_j_ × *T*_k _= interaction between BCS class and treatment; *C*_j_ × *W*_l _= interaction between BCS class and time; *T*_k_ × *W*_l _= interaction between treatment and time; *C*_j_ × *T*_k _× *W*_l_ = interaction between BCS class, treatment and time; and ε_i·l_ = residual error (or cow × time). The necessity of an auto‐regressive function and heterogeneity were tested and judged by the difference in deviance of each model with the change in degrees of freedom in a chi‐squared distribution. Auto‐regressive function improved model deviance of all parameters; heterogeneity was not always relevant but for most parameters outside the model, as displayed with the model components in Table S1. A visual check for outliers was performed based on model residuals after which the model was tested with and without the potential outlier to see if conclusions changed; this was not the case, and no outliers were deleted.

The significance value was set at *p* < .05, and a trend was declared at 0.05 ≤ *p *< .10.

## 3 RESULTS

Thirty cows finished the experiment. Two cows were excluded, one cow due to a hip injury after an accident not related to the experimental procedures (group CON) and one cow aborted just before the start of the experiment (group STEP).

### 3.1 Dry period exercise

**Figure 1:**
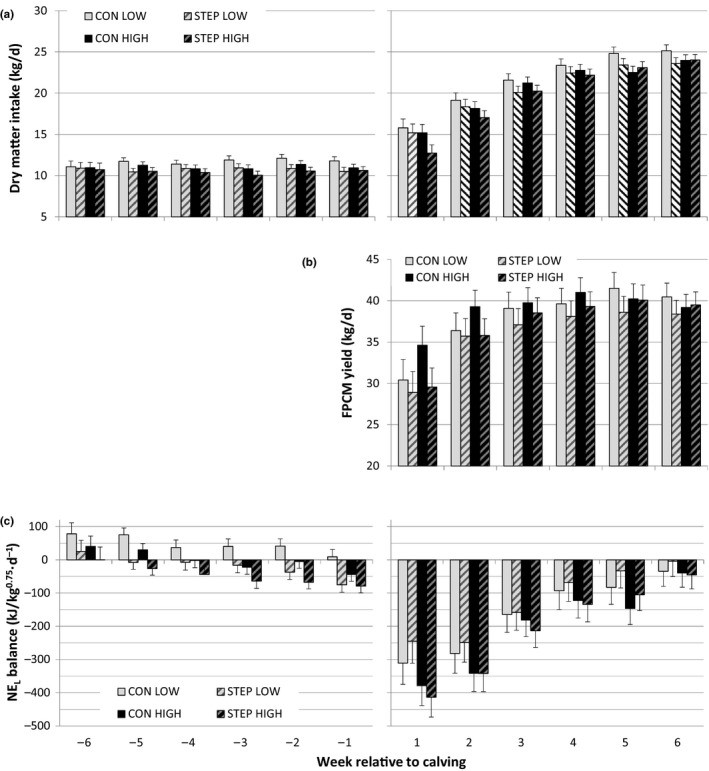
Cow performance regarding (a) average dry matter intake (kg/day); (b) fat‐ and protein‐corrected milk yield (FPCM yield, kg/day); and (c) calculated net energy for lactation (NE_L_) balance (kJ/kg^0.75^·day^−1^) during the experiment from 6 weeks before until 6 weeks after calving. Figures showing predicted means by REML analysis with *SEM*

During the dry period, BW increased on average by 27 kg, but there was no difference between treatment groups. The average BW during the dry period was 679 vs. 705 kg for STEP and CON respectively (SED = 19.7 and *p* = .118). As expected, cows in STEP group had a lower NE_L_ balance (−33 vs. +23 kJ/kg^0.75^·day^‐1^ for STEP and CON, respectively, SED = 18.0 and *p* < .01). Energy balance was below zero from 5 weeks before calving while CON cows had an energy balance below zero from 1 week before calving (Figure 1). Exercise affected feed intake: prepartum DM intake (DMI) was 0.8 kg DM/day lower for STEP relative to CON cows (10.6 vs. 11.4 for STEP and CON, respectively, SED = 0.37 and *p* < .05).

During the prepartum training period, cows in BCS class HIGH had lower plasma cholesterol concentration (3.5 vs. 4.1 mmol/L, SED = 0.25 and *p* < .05) compared to LOW without a difference in NEFA or BHB; cows in group STEP showed significantly higher plasma NEFA (0.67 vs. 0.25 mmol/L, SED = 0.101 and *p* < .001) and BHB concentration (0.69 vs. 0.46 mmol/L, SED = 0.076 and *p* < .01) relative to cows in group CON.

### 3.2 Postpartum performance

Treatment or BCS class did not affect average gestation length (280 days) or birth weight of calves (44 kg). After calving, feed intake increased over six weeks from 15 to 24 kg DM per day, milk yield from 26 to 40 kg per day and the NEB slowly increased towards zero (Figure 1); average body weight and BCS are shown in Table 3. Neither DMI, milk yield nor NE_L_ balance was affected by exercise, BCS class or their interaction (Table 4). There was however a tendency for a reduced DMI in BCS class HIGH in the first two weeks after calving (interaction week × BCS, SED = 0.66 and *p* = .071) as well as a tendency for the interaction of week × exercise ×BCS in FPCM production (SED = 2.40 and *p* = .053), due to the high FPCM yield in week 1 for non‐exercised cows in BCS class HIGH (Table 4, Figure 1).

**Table 3:** Descriptive statistics for body weight in kg (BW) and body condition score on a scale from 1 to 5 (BCS) at start of the trial (6 weeks before calving), directly after calving and at the end of the experimental period (week 6 postpartum)

	Start	After calving	End
Body weight			
LOW CON	662 (66)	631 (53)	647 (56)
LOW STEP	641 (68)	603 (58)	616 (55)
HIGH CON	713 (52)	677 (62)	660 (59)
HIGH STEP	719 (63)	643 (43)	662 (40)
Body condition score			
LOW CON	2.9 (0.4)	3.1 (0.5)	2.7 (0.9)
LOW STEP	2.8 (0.6)	2.5 (0.3)	2.3 (0.5)
HIGH CON	3.7 (0.5)	3.1 (0.4)	2.8 (0.6)
HIGH STEP	3.7 (0.3)	3.0 (0.3)	2.8 (0.4)

Note

For each treatment group, the average and standard deviation (between brackets) are shown (*n* = 8 per group).

**Table 4:** The effect of prepartum BCS class (HIGH vs. LOW) and exercise (CON vs. STEP) on dry matter intake (DMI), milk yield, fat‐ and protein‐corrected milk (FPCM), milk components (fat, protein, lactose), component yield and calculated net energy for lactation balance (NE_L_ bal) in week 1 to 6 postpartum

Item	Treatment group				SED	P‐value^a^					
	Low con (*n* = 7)	Low step (*n* = 7)	High con (*n* = 8)	High step (*n* = 8)		Exerc	BCS	Exerc × BCS	Exerc × week	BCS × week	Exerc × BCS ×week
DMI (kg/day)	21.7	20.5	20.7	19.9	0.97	0.184	0.556	0.485	0.654	0.071	0.383
Milk yield (kg/day)	35.7	35.2	36.3	35.2	3.03	0.905	0.905	0.831	0.195	0.773	0.150
FPCM (kg/day)	37.9	36.2	39.0	37.2	2.59	0.990	0.990	0.532	0.765	0.363	0.053
Milk fat (g/kg)	45.3	42.8	47.3	45.6	1.94	0.045	0.548	0.745	0.175	<0.001	0.735
Milk protein (g/kg)	36.5	34.1	34.2	34.9	1.87	0.840	0.498	0.086	0.873	0.936	0.963
Milk lactose (g/kg)	46.0	46.4	45.5	46.1	0.56	0.135	0.523	0.582	0.107	0.083	0.134
Fat yield (kg/day)	1.58	1.49	1.68	1.56	0.130	0.289	0.974	0.799	0.892	0.106	0.218
Protein yield (kg/day)	1.27	1.17	1.22	1.20	0.101	0.954	0.892	0.252	0.282	0.451	0.033
Lactose yield (kg/day)	1.64	1.63	1.66	1.64	0.128	0.775	0.850	0.721	0.154	0.744	0.238
NE_L_ bal (kJ/kg^0.75^·day^−1^)	−161	−126	−202	−209	68.2	0.885	0.672	0.656	0.380	0.154	0.925

Abbreviations: BCS, body condition score class (LOW vs. HIGH); Exerc, exercise group (CON vs. STEP).

a The main effect “week” was significant for all parameters reported (*p* < .001).

Cows with BCS class HIGH had initially greater milk fat content compared with LOW (interaction week × BCS, SED = 1.49 and *p* < .001; Table 4 or Figure S1) while the average milk fat content postpartum was reduced for treatment STEP compared with CON (44.2 vs. 46.3 g/kg, SED = 1.37 and *p* < .05; Table 4). Fat yield in kg per day was not affected by exercise, BCS class or their interaction and neither were protein content, lactose content or lactose yield. Protein yield showed a significant interaction of week × exercise ×BCS (SED = 0.09 and *p* < .05; Table 4).

**Figure 2:**
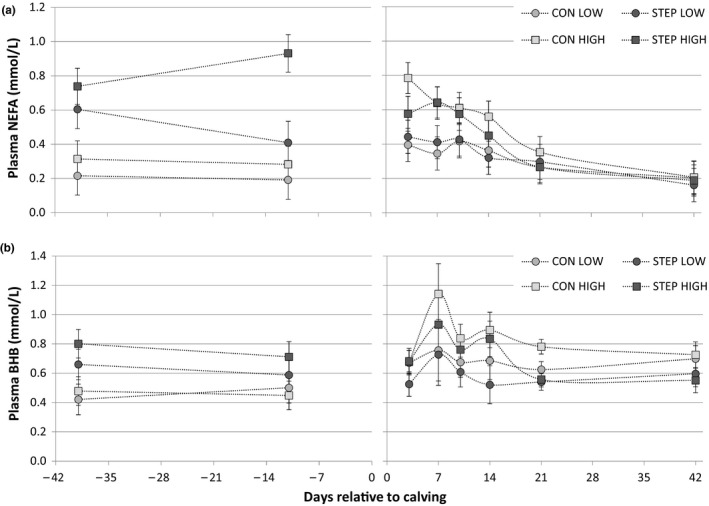
Fatty acid metabolism as measured by concentration of (a) plasma non‐esterified fatty acids (NEFA, mmol/L) and (b) plasma β‐hydroxybutyric acid (BHB, mmol/L). Figures showing predicted means by REML analysis with *SEM*

Postpartum NEFA concentration was affected by prepartum BCS: cows in BCS class HIGH had higher NEFA concentration compared with LOW (0.49 vs. 0.34 mmol/L, SED = 0.061 and *p* < .05, Table 5, Figure 2a). Concentration of NEFA was not affected by treatment, but plasma BHB was on average lower for treatment STEP compared with CON (0.65 vs. 0.76 mmol/L, SED = 0.068 and *p* < .01, Table 5, Figure 2b).

**Table 5:** The effect of prepartum BCS class (HIGH vs. LOW) and exercise (CON vs. STEP) on average plasma concentrations of non‐esterified fatty acids (NEFA), β‐hydroxybutyric acid (BHB), total cholesterol, high‐density lipoprotein cholesterol (HDL‐chol), low‐density lipoprotein cholesterol (LDL‐chol) and very low‐density lipoprotein (VLDL‐chol) postpartum (determined on 3, 7, 10, 14, 21 and 42 days after calving), all in mmol/L; and liver TG determined in 16 cows in week −2, week 1 and week 2 relative to calving (expressed in g/kg wet weight (ww) and as a relative increase compared to week −2)

Item	Treatment group				SED	P‐value^a^					
	LOW CON	LOW STEP	HIGH CON	HIGH STEP		Exerc^b^	BCS^c^	Exerc × BCS	Exerc × time	BCS × time	Exerc × BCS ×time
	(*n* = 7)	(*n* = 7)	(*n* = 8)	(*n* = 8)							
NEFA (mmol/L)	0.33	0.34	0.53	0.45	0.086	0.535	0.018	0.474	0.909	0.206	0.888
BHB (mmol/L)	0.69	0.59	0.84	0.72	0.096	0.009	0.127	0.493	0.891	0.477	0.500
Cholesterol (mmol/L)	2.94	2.94	3.01	2.70	0.280	0.416	0.440	0.460	0.039	0.272	0.498
HDL‐chol (mmol/L)	2.08	2.07	2.12	1.93	0.181	0.586	0.650	0.912	0.059	0.196	0.713
LDL‐chol (mmol/L)	0.84	0.85	0.86	0.72	0.104	0.504	0.142	0.372	0.397	0.813	0.548
VLDL‐chol (mmol/L)	0.02	0.02	0.04	0.04	0.007	0.957	0.001	0.802	0.808	0.744	0.339
	(*n* = 4)	(*n* = 4)	(*n* = 4)	(*n* = 4)							
Liver TG (g/kg ww)	17	21	53	36	10.3	0.499	0.005	0.160	0.137	0.007	0.331
Liver TG (relative)	1.8	1.7	3.9	2.3	0.71	0.099	0.058	0.229	0.202	0.043	0.359

Abbreviations: Exerc, exercise group (CON vs. STEP); BCS, body condition score class (LOW vs. HIGH).

a The main effect “time of sampling” was significant for all parameters reported (*p* < .001), except for BHB (tendency, *p* = .089) and VLDL‐chol (*p* = .235).

^b,c^ The significance value was set at *p* < .05, and a trend was declared at 0.05 ≤ *p* < .10.

### 3.3 Liver metabolism and lipoprotein transport

**Figure 3:**
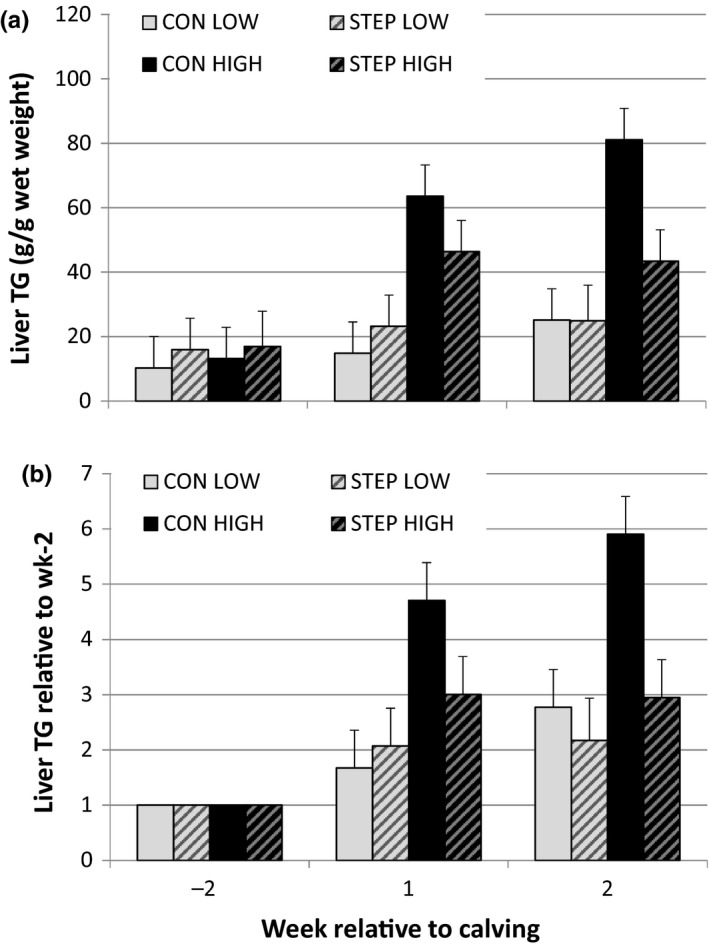
Liver triglyceride (TG) concentration for the subset of 16 cows with liver biopsies taken at week −2, week 1 and week 2 relative to calving with (a) absolute TG concentration, in g/kg wet weight; and (b) TG concentration expressed relative to week −2. Figures showing predicted means by REML analysis with *SEM*

Liver TG concentration increased after calving and was affected by prepartum BCS. Cows in BCS class HIGH showed a greater increase in liver TG compared with LOW (BCS × time, SED = 9.3 and *p* < .01, Table 5, Figure 3a). Liver TG was not affected by prepartum exercise. The relative increase in liver TG postpartum compared with liver TG in week −2 tended to be lower in cows in group STEP compared with CON (2.1 vs. 2.8, SED = 0.50 *p* = .099, Table 5, Figure 3b).

Prepartum cholesterol was not affected by exercise. During prepartum, concentrations of plasma cholesterol were lower for cows in BCS class HIGH compared with LOW (3.5 vs. 4.1 mmol/L, SED = 0.25 and *p* < .05). At 3 days postpartum, plasma cholesterol was relatively low and not affected by BCS class, but the increase over time was reduced for cows in group STEP compared with CON (interaction week × treatment, SED = 0.18 and *p* < .05, Table 5, Figure S2).

The subgroups of HDL and LDL were not affected by BCS class or exercise postpartum, with a tendency for the interaction of week × exercise for HDL cholesterol (SED = 0.118 and *p* = .059). Concentration of VLDL cholesterol was extremely low, but higher for BCS class HIGH compared with LOW (SED = 0.005 and *p* < .01) as shown in Table 5 and Figure S2.

## 4 DISCUSSION

Our hypothesis that increasing physical activity will start fat mobilization prepartum is supported by the results of this proof of concept trial, which may reduce the risk for hepatic lipidosis in cows with a high BCS at dry‐off. As expected, the increase in hepatic TG content postpartum was highest for cows in BCS class HIGH. The relative increase postpartum compared with week −2 prepartum tended to be reduced by exercise. This may be explained by a difference in plasma lipoprotein transport, as VLDL is presumed to be the main export route of TG from the liver, returning to the liver as LDL (Van Den Top et al., 1995; Zom et al., 2011; Newman, Mann, Nydam, Overton, & Behling‐Kelly, 2016). Exercise did however not affect lipoprotein transport in the current trial. Lipid transportation by VLDL/LDL lipoproteins was low in plasma for all treatment groups after calving compared with their concentrations pre‐calving. A reduction in VLDL and LDL cholesterol concentration immediately after calving when plasma NEFA concentrations are greater has also been reported by others (Gross, Kessler, Albrecht, & Bruckmaier, 2015; Newman et al., 2016). Low lipid transport seems to be conflicting with the increased lipolysis early postpartum. This may be attributed to the homeorhetic changes in metabolism peripartum, such as the high priority for milk synthesis resulting in an increased utilization of lipids by the mammary gland. In a later stage of lactation, the metabolic adaptation changes in that feed restriction will directly result in increased VLDL and LDL cholesterol concentrations, in contrast to the situation in the periparturient period in the same cows (Gross et al., 2015).

To our knowledge, the effects of exercise on lipoprotein metabolism or hepatic fat accumulation have not been studied before in dairy cattle. Initiation of lipolysis antepartum was expected to induce fat transport prepartum, earlier for exercised cows compared with non‐exercised. This did however not affect plasma lipoprotein concentration as expected. The postpartum relative accumulation of hepatic fat of exercised cows tended to be lower, while the magnitude of lipolysis was comparable to non‐exercised cows (no difference in plasma NEFA concentration postpartum). It may be hypothesized that the processing of lipids must have been improved in liver or in peripheral tissue. The actual rate of lipid transport from the liver may be underestimated by measuring plasma lipoprotein cholesterol, as this concentration represents the balance between liver excretion and peripheral uptake or utilization. Exercising cows prepartum likely induced lipolysis, increased lipid uptake and lipoprotein turnover, priming energy metabolism as suggested by Friggens et al. (2004) and could thereby reduce the risk for extreme hepatic fat accumulation in the first weeks postpartum. In humans, exercise reduces intrahepatic liver accumulation through improved NEFA uptake in skeletal muscle and reduced NEFA uptake in liver without a change in VLDL secretion (Brouwers, Hesselink, Schrauwen, & Schrauwen‐Hinderling, 2016).

Feed intake of STEP cows was reduced prepartum, which was not attributable to a difference in access time to the feed mixture in that feeding bins were inaccessible to all cows (STEP and CON) during training sessions. In a trial with heifers, forced exercise for 1.6 km/day until calving did not result in differences in feed intake; energy intake did reduce when forced exercise was continued for the first 10 days post‐calving (Lamb, Barker, Anderson, & Walters, 1979). In contrast, forced exercise in multiparous animals for 3.2 or even 9.7 km/day did not affect prepartum DMI significantly (Anderson et al., 1979). In a study with tied dairy cows, daily exercise of 2–3 km/day did not affect voluntary feed intake (Gustafson, 1993). The amount of exercise in the present trial was however greater and also involuntary, which may have reduced feed intake through stress. Exercise also increases heat production and rectal temperature in ruminants (Piguet, Bruckmaier, & Blum, 1994), which might be hypothesized to result in some degree of heat stress, thereby reducing feed intake (Koch, Lamp, Eslamizad, Weitzel, & Kuhla, 2016). Unfortunately, water intake was not measured in this trial but seemed to be increased directly after the training session upon return in the barn, especially on warm and sunny days (personal observation). This is suggestive for some degree of heat stress and could also have affected feed intake.

The forced physical activity in combination with lower feed intake prepartum resulted in a negative net energy balance from 5 weeks before calving in cows in the STEP group. Prolonged exercise by itself can also increase lipolysis and NEFA utilization in muscle as studied in sheep, where a walking speed of 4.5 km/hr increased plasma NEFA from 0.1 to 0.9 after 45 min (Pethick, Harman, & Chong, 1987). These effects match with the increased concentration of plasma NEFA in cows in the STEP group before calving and further increasing towards calving for the cows with body condition HIGH in the STEP group, indicating increased lipolysis. This concept may seem contradictory to results from field studies on dairy farms, where an increased NEFA concentration prepartum (>0.3 mmol/L) was found to be a risk factor for postpartum disease (Chapinal et al., 2011). However, in our study NEFA concentration (and BHB concentration) was increased in healthy cows by physical activity, while cows in field studies with high NEFA prepartum may (a) already suffer from health problems before calving and (b) have less metabolic capacity to start adaptation of fat metabolism sufficiently.

Postpartum, the calculated energy balance was negative for all cows, also indicated by a decreasing BCS, while DMI and milk yield level were comparable between groups. Cows in BCS class HIGH produced milk with a greater fat content, especially in the first weeks postpartum. High BCS is directly related to increased mobilization of adipose tissue and high milk fat excretion (Rukkwamsuk, Wensing, & Kruip, 1999), which was also confirmed in our study. HIGH cows had increased plasma NEFA concentrations during early postpartum compared with LOW cows. We found no effect of exercise on NEFA concentration postpartum. However, exercised cows (group STEP) had lower plasma BHB and lower milk fat content after calving, which suggests a carryover effect of prepartum exercise on lipid mobilization and processing in early lactation. This reduction in milk fat postpartum is similar to results reported with multiparous animals subjected to prepartum exercise (Anderson et al., 1979), but not with heifers (Lamb et al., 1979).

The application of forced exercise for dry cows as used in this study is not immediately feasible on dairy farms, due to time and labour commitments. Simply providing access to pasture does not directly stimulate cows to increase their activity sufficiently compared with forced exercise (Black, Van Amstel, & Krawczel, 2017). Cows will still need to be stimulated to exercise, for example by a daily return walk between barn and paddock, or by providing water and feed at different places in the exercise area.

## 5 CONCLUSIONS

A substantial amount of physical exercise in the dry period in this study affected fat metabolism of dairy cows in the prepartum period, and these effects carried over into the postpartum period. Exercise can induce lipid mobilization and utilization prepartum and may thereby start metabolic adaptation of dairy cows before the onset of milk production. Other adaptive mechanisms related to the carbohydrate metabolism or endocrine regulation may also be involved, which were not measured in this study. The hypothesis that physical activity prepartum will reduce the risk on excessive fat mobilization postpartum is most promising for cows with a greater BCS at dry‐off. Further research is needed to confirm our results in a larger group of animals and to improve our understanding of the underlying physiological pathways.

## CONFLICT OF INTEREST

The authors have no conflict of interest to declare.

## ANIMAL WELFARE STATEMENT

The authors confirm that the ethical policies of the journal, as noted on the journal's author guidelines page, have been adhered to and the appropriate ethical review committee approval has been received. The authors confirm that they have followed EU standards for the protection of animals used for scientific purposes.
