# Supplementary material for: Physical exercise prepartum to support metabolic adaptation in the transition period of dairy cattle: A proof of concept
Source: J Anim Physiol Anim Nutr (Berl). 2020 Feb 25;104(3):790–801. doi: 10.1111/jpn.13330 (PMC7217059; doi:10.1111/jpn.13330)
Supplement: Supplementary file 1 [file JPN-104-790-s001.pdf]

SUPPORTING INFORMATION

Physical exercise prepartum supports metabolic adaptation in the transition period of dairy cattle

R. M. A. Goselink, J. T. Schonewille, G. van Duinkerken, and W. H. Hendriks

Table S1 Model characteristics and variance components of the random coefficient model analysis of each parameter by REML method.

	Deviance (df)	Variance components		Hetero-geneity	Variance component					
		Cow	Time		Cow × Time					
Liver TG										
g/kg wet weight	249 (32)	120.7	-	None	256.2					
relative to wk-2	67 (32)	-5.5	0.86	None	7.39					
Prepartum					*wk-6*	*wk-5*	*wk-4*	*wk-3*	*wk-2*	*wk-1*
DMI (kg/d)	230 (142)	0.74	0.22	Outside	2.61	0.64	0.85	1.00	0.82	0.99
NE_L bal ($kJ/kg^{0.75}\cdot d^{-1}$)	1130 (118)	1576.4	0.26	Outside	5030	978	1634	1498	1269	1413
NEFA (mmol/L)	-67 (47)	0.045	0.34	None	0.34					
BHB (mmol/L)	-62 (47)	0.038	-0.85	None	0.038					
Cholesterol (mmol/L)	18 (47)	0.27	0.41	None	0.27					
HDL-chol (mmol/L)	-38 (47)	0.069	-0.07	None	0.069					
LDL-chol (mmol/L)	-47 (47)	0.080	0.56	None	0.080					
VLDL-chol (mmol/L)	-248 (47)	0.001	0.097	None	0.001					
Postpartum					*wk1*	*wk2*	*wk3*	*wk4*	*wk5*	*wk6*
DMI (kg/d)	306 (146)	2.3	0.63	Outside	5.9	3.3	1.9	2.0	2.0	1.4
Milk yield (kg/d)	415 (146)	32.8	0.56	Outside	11.0	3.4	1.5	4.1	1.6	1.6
FPCM (kg/d)	446 (144)	19.1	0.81	Outside	23.5	12.7	8.0	5.8	7.0	0.7
Milk fat (g/kg)	584 (144)	10.3	0.39	Outside	22.7	11.3	10.9	11.8	3.9	13.8
Milk protein (g/kg)	362 (144)	9.3	0.82	Outside	13.6	4.8	3.3	1.7	13.9	1.0
Milk lactose (g/kg)	131 (144)	0.97	0.37	Outside	1.41	0.70	0.31	0.35	0.90	0.09

Fat yield (kg/d)	-411 (144)	0.043	0.80	Outside	0.072	0.050	0.032	0.022	0.030	0.000
Protein yield (kg/d)	-529 (144)	0.027	0.83	Outside	0.035	0.017	0.012	0.005	0.031	0.004
Lactose yield (kg/d)	-491 (144)	0.057	0.60	Outside	0.027	0.011	0.007	0.012	0.007	0.000
NE_L bal ($kJ/kg^{0.75} \cdot d^{-1}$)	1280 (121)	12248	0.61	Outside	12545	8783	5343	7103	3550	240
NEFA (mmol/L)	-236 (147)	0.015	0.21	None	0.050					
BHB (mmol/L)	-220 (142)	0.008	0.35	Outside	0.040	0.300	0.065	0.110	0.013	0.048
Cholesterol (mmol/L)	-150 (142)	0.20	0.69	Outside	0.086	0.053	0.055	0.116	0.180	0.718
HDL-chol (mmol/L)	-256 (142)	0.072	0.75	Outside	0.002	0.035	0.050	0.098	0.154	0.237
LDL-chol (mmol/L)	-346 (142)	0.021	0.58	Outside	0.024	0.007	0.012	0.026	0.053	0.207
VLDL-chol (mmol/L)	-913 (147)	0.0001	0.076	None	0.001					

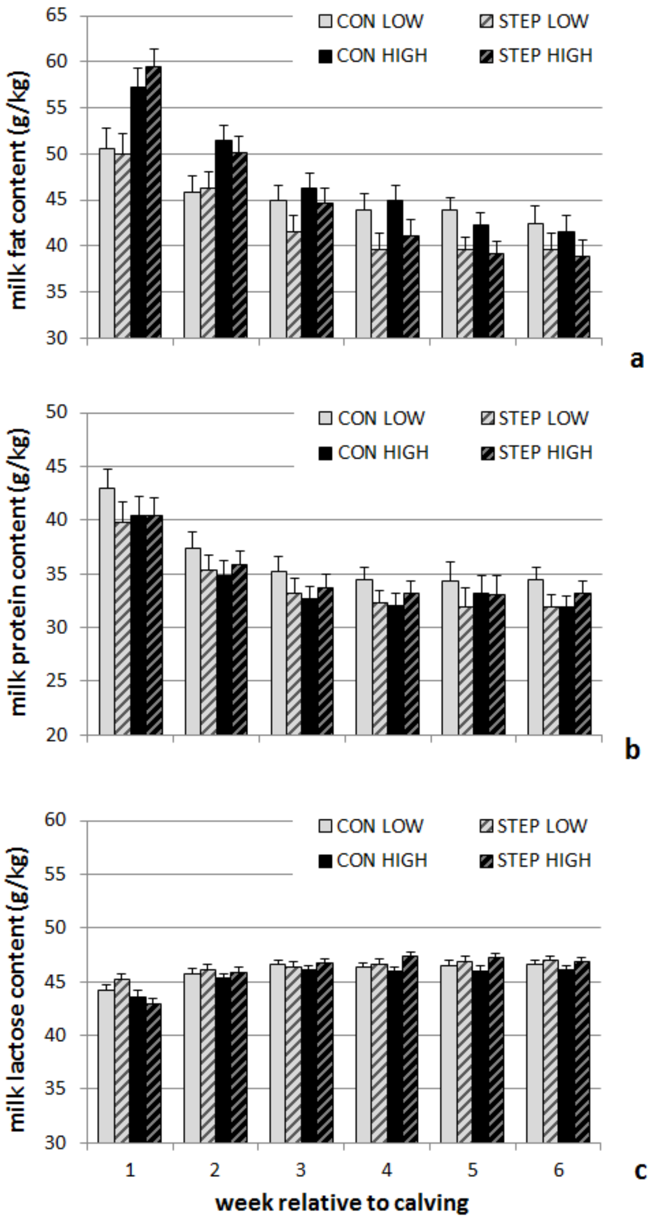

Figure S1 Average milk content (g/kg) of a) fat; b) protein; and c) lactose for each treatment group during the experiment. Figures showing predicted means by REML analysis with SEM.

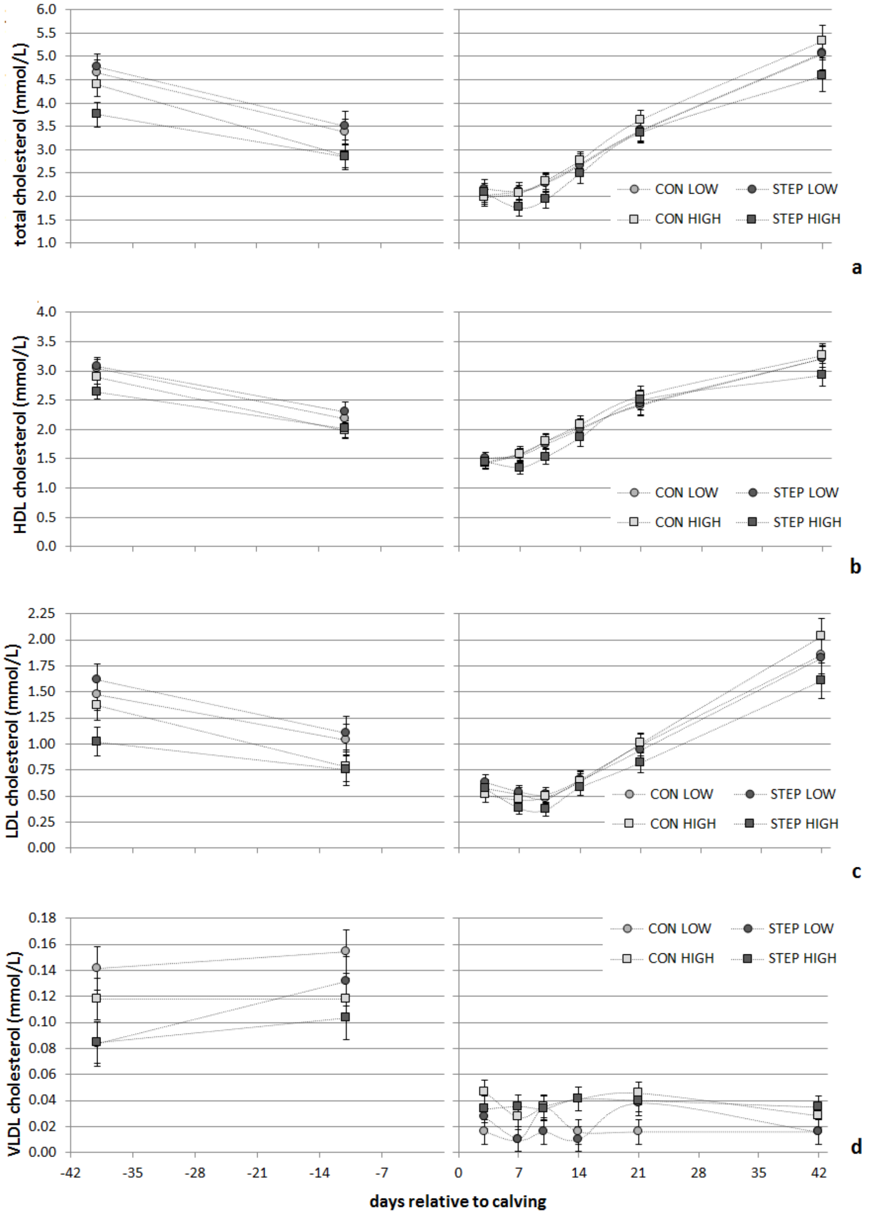

Figure S2 Average plasma concentration (in mmol/L) of a) total cholesterol; b) HDL cholesterol; c) LDL cholesterol; and d) VLDL cholesterol. Figures showing predicted means by REML analysis with SEM.
